# Examining the relationship between body mass index and adverse cardio-metabolic profiles among Australian Indigenous and non-Indigenous young adults

**DOI:** 10.1038/s41598-019-40083-x

**Published:** 2019-03-04

**Authors:** Arusyak Sevoyan, Belinda Davison, Alice Rumbold, Vivienne Moore, Gurmeet Singh

**Affiliations:** 1 School of Public Health, University of Adelaide, Adelaide, SA Australia; 2 Robinson Research Institute, University of Adelaide, Adelaide, SA Australia; 3 Menzies School of Health Research, Casuarina, NT 0811 Australia; 4 School of Medicine, University of Adelaide, Adelaide, SA Australia

## Abstract

Australian Indigenous young people have a 10-fold excess of deaths from ischaemic heart disease compared with non-Indigenous Australians, yet the reasons behind this remain understudied. This paper aims to describe cardio-metabolic profiles among Australian Indigenous (n = 459) and non-Indigenous (n = 117) young adults (21–27 years). The association between body size and an adverse cardio-metabolic profile (≥3 abnormal cardio-metabolic markers) is assessed by gender and urban/rural residence, employing regression analyses. The prevalence of obesity was highest among urban Indigenous participants, both males and females (22% and 23%, respectively). Overall, BMI showed a significant positive association with an adverse cardio-metabolic profile. Moreover, adverse cardio-metabolic profile was present in a substantial proportion of Indigenous participants even in overweight and normal BMI categories. Among females, this could reflect elevated waist circumference, which was present in half of those of normal weight. Remote Indigenous females had the highest predicted probability of having an adverse cardio-metabolic profile across all BMI categories (13% for underweight, 54% for normal BMI, 93% for overweight, and 99% for obese). Our findings highlight the associations between obesity and adverse cardio-metabolic profiles among Indigenous and non-Indigenous youth. Culturally-relevant strategies that address lifestyle risks, including access to healthy food, are urgently needed in this age group.

## Introduction

Cardiovascular disease (CVD) is the leading cause of morbidity and mortality, globally^1^. There is evidence that susceptibility to CVD originates in early life, as biological risk factors, such as hypertension, dyslipidaemia and insulin resistance, identified in childhood track into adulthood^2,3^, when they may be exacerbated by behavioural risk factors such as smoking, alcohol consumption and lack of physical activity. Biological risk factors of CVD tend to cluster, partly due to the influence of obesity as it affects a range of metabolic and physiological processes, and thereby, biological risk factors for CVD^4^.

Australia’s Aboriginal and Torres Strait Islander populations (hereafter referred to as Indigenous) experience disproportionately high rates of CVD, particularly in the younger age groups^5^. Premature deaths from CVD are the largest contributor to the 10 year gap in life expectancy between Indigenous and non-Indigenous Australians^6^. The reasons for the high burden of CVD among Indigenous people are complex, and include a high prevalence of risk behaviours, poorer access to primary and tertiary health care, including a lack of culturally appropriate care, as well as upstream factors, such as social disadvantage and racism, which have been experienced for many generations^7^.

Obesity, defined as having a body mass index (BMI) of 30 or more^8^, is a significant health issue for Indigenous people, with 42% of Indigenous adults affected with obesity compared with 27% of non-Indigenous adults^9^. Among young people aged 18–24 years, the prevalence is 27% and 14%, respectively^9^. Overweight and obesity are associated with a significant reduction in life expectancy, to a large extent through the contribution to CVD^1,10^. Moreover, preliminary evidence indicates that an earlier onset and longer duration of obesity in childhood are associated with increased cardio-metabolic risk in adolescence^11^.

Whilst previous studies have demonstrated an increased prevalence of cardiovascular risk factors, such as obesity, dyslipidaemia, hypertension and diabetes, in Indigenous compared to non-Indigenous adults^12–14^, there is a paucity of information in young adulthood. This paper aims to provide information on the cardio-metabolic profiles of young Indigenous and non- Indigenous adults in the Northern Territory (NT), Australia and explores the impact of Indigeneity, place of residence and gender. It also examines the associations between BMI and adverse cardio-metabolic profiles in this high risk cohort using data from the Life Course Program.

## Methods

The Life Course Program is a prospective longitudinal study examining effects of early life factors on later health and disease in Indigenous and non-Indigenous Australians. It encompasses two distinct but complementary cohorts: the Aboriginal Birth Cohort (ABC) and the non-Indigenous Top End Cohort (TEC). Participants of both the ABC and TEC (459 and 117, respectively) were examined in young adulthood (aged 21–27 years) between August 2013 and June 2015 in their community of residence.

The recruitment and previous follow up of the ABC^15^ and TEC^16^ studies have been described elsewhere. In brief, between 1987 and 1990, 686 (54% of eligible) babies born to Indigenous mothers were recruited from the Royal Darwin Hospital, the main referral hospital for the NT, to the ABC study^15,17^. Subsequent follow-up has occurred at the participant’s residence in over 40 urban and remote communities across the NT at mean age of 11 years (86% examined)^18^, and mean age of 18 years (71% examined). Between 2007 and 2009, 196 adolescents born to non-Indigenous mothers in Darwin between 1987 and 1991, age matched to participants of the ABC study, were recruited to the TEC study^16^.

### Measurements

Assessments were conducted by a trained researcher using standardised techniques. Participants were measured in light clothing and barefoot. Height was measured to the nearest millimetre using a portable calibrated wall mounted stadiometer (MZ10017, ADE GmbH & Co., Germany). Weight was measured to 0.1 kg with a digital electronic scale (TBF-521, Tanita Corporation, Illinois, USA). Body mass index (BMI) (kg/m^2^) was calculated and categorised as underweight <18.5, normal 18.5–24.9, overweight 25–29.9 and obese ≥30^8^. Flexible tapes were used to measure waist circumference in a horizontal plane, midway between the lowest ribs and the iliac crests at the end of a gentle expiration^19^.

Sitting blood pressure was measured, after 5–10 minute rest, three times on the right arm, using an appropriate cuff size (Welch Allyn Lifesigns monitor). The mean of the three measurements was used for analysis.

Venous blood was obtained from fasting and non-fasting participants with whole blood frozen for estimation of glycated haemoglobin (HbA1c), and serum and plasma obtained from blood centrifuged post clotting within <2 hours of collection and frozen. Frozen status was maintained throughout storage and transportation to testing laboratories.

High-density lipoprotein cholesterol (HDL-c) and triglycerides were measured by Enzymatic colorimetric assay (Siemens XP and Plus), high-sensitive C-reactive protein (hs-CRP) was measured by Immuno-turbidimetric (Randox/Siemens XP and Plus). Glycated haemoglobin concentration (HbA1c) was measured by Immunoassay (Siemens XP and Plus).

Basic socio-economic background information collected from the participants included years of schooling (whether or not completed 12 years), marital status (single/divorced or married/de facto), car ownership (whether or not owned a car, someone in the household owned a car or there was no car in the household), and the main source of household income (whether or not the main source of household income was employment). Details on exercise (none, <5 hours, 5–10 hours, >10 hours per week), smoking status (current smoker vs. non-smoker/ex-smoker), and alcohol consumption (occurrence and frequency) were self-reported as part of a lifestyle questionnaire.

### Adverse cardio-metabolic profile

Adverse cardio-metabolic profile is defined as the presence of three or more abnormal cardio-metabolic markers, including elevated waist circumference (≥94 cm for males and ≥80 cm for females)^20^; elevated triglycerides or drug treatment for elevated triglycerides (≥150 mg/dL (1.7 mmol/L)^20^; reduced HDL-c or drug treatment for reduced HDL-c (<40 mg/dL (1.0 mmol/L) in males; <50 mg/dL (1.3 mmol/L) in females)^20^; elevated blood pressure (systolic ≥130 and/or diastolic ≥85 mm Hg) or antihypertensive drug treatment in a patient with a history of hypertension^20^; elevated HbA1c (≥5.7% (39 mmol/mol)), which has been suggested to be a useful alternative to fasting plasma glucose (FPG) in identifying metabolic syndrome due to its strong agreement with FPG^21–25^; and elevated hs-CRP (≥3 μg/mL)^26^, which has been identified as a powerful indicator adding to the predictive value of metabolic syndrome in CVD prognosis and as an independent factor associated with incident coronary heart disease^27,28^.

### Statistics

Descriptive statistics for demographic, socio-economic and lifestyle characteristics were generated for subgroups of Indigeneity/residence and gender. The area of residence was classified as ‘remote’ (living in a rural community with an Aboriginal council) and ‘urban’ (living in Darwin and its surrounding areas)^29^. It must be noted that the ‘remote’ category included only Indigenous participants, while all non-Indigenous TEC participants were from ‘urban’ areas. For this reason we use 3 combined Indigeneity/residency categories throughout our analyses, including Remote Indigenous, Urban Indigenous and Urban non-Indigenous.

Cardio-metabolic measures were dichotomised, using the abnormal cut-off points defined above, to create categories of normal and abnormal, and their associations with BMI categories were assessed using the Pearson Chi-square test. Differences were considered statistically significant at *p* < 0.05.

Multivariate logistic regression was used to assess the net effect of BMI on adverse cardio-metabolic profile with adjustment for Indigeneity/residence, gender and socio-economic status, including: years of schooling, car ownership and the main source of household income. For these analyses, BMI was considered as a continuous variable. All other explanatory variables were treated as categorical. Subsequent models were created that controlled for lifestyle variables, including exercise, alcohol intake and smoking. To test for the moderating effect of gender on the associations between the adverse cardio-metabolic profile and Indigeneity/residency we have included these interaction terms in the analysis. The results are presented in separate models for males and females for the ease of interpretability. All the cases with missing data were excluded from multivariate analysis. Pregnant women (n = 26) were excluded from both bivariate and multivariate analyses. All the statistics were computed using SAS 9.3.

### Ethical Standards

This study was approved by the Human Research Ethics Committee of NT Department of Health and Menzies School of Health Research, including the Aboriginal Ethical Sub-committee which has the power of veto (ABC Reference no. 2013-2022 and TEC Reference no. 2013–1986). All research was performed in accordance with the National Health and Medical Research Council guidelines^30^. Informed written consent was obtained from all participants.

## Results

Substantial differences in the sociodemographic profiles were seen between Indigenous and non-Indigenous participants (see Table 1). The majority of urban non-Indigenous participants had 12 years of schooling, whilst only about 20–30% of urban and remote Indigenous participants had completed 12 years of schooling. Almost all urban non-Indigenous males reported employment as the main source of household income, compared with under half of urban and remote Indigenous males. All groups of females were significantly more likely than their male counterparts to depend on Social Support as the main source of income, although the majority of urban non-Indigenous females reported employment as their main source of income.

**Table 1:** Demographic, socio-economic, lifestyle characteristics and BMI of Life Course Program wave 4 study participants, by Indigeneity, residence and gender.

	Males (n = 259)			Females (n = 317)		
	Remote Indigenous (n = 160)	Urban Indigenous (n = 56)	Urban non-Indigenous (n = 43)	Remote Indigenous (n = 191)	Urban Indigenous (n = 52)	Urban non-Indigenous (n = 74)
Age	25 [25, 26]	26 [25, 26]	24 [23, 25]	25 [24, 26]	26 [25, 27]	24 [22, 25]
Years of schooling						
<12 years	79.4% (127)	74.5% (41)	9.5% (4)	69.9% (130)	73.1% (38)	2.7% (2)
12 years	20.6% (33)	25.5% (14)	90.5% (38)	30.1% (56)	26.9% (14)	97.3% (71)
Marital status						
Single/Divorced	32.5% (52)	42.6% (23)	72.1% (31)	37.7% (72)	42.3% (22)	54.0% (40)
Married/De Facto	67.5% (108)	57.4% (31)	27.9% (12)	62.3% (119)	57.7% (30)	46.0% (34)
Main source of household income						
Employment	36.5% (58)	48.2% (27)	95.4% (41)	15.2% (29)	27.5% (14)	86.5% (64)
Social Support/other	63.5% (101)	51.8% (29)	4.6% (2)	84.8% (162)	72.5% (37)	13.5% (10)
Car ownership						
No car in the household	46.9% (75)	25.0% (14)	2.3% (1)	45.0% (86)	32.7% (17)	4.0% (3)
Owns a car	23.8% (38)	44.6% (25)	90.7% (39)	17.3% (33)	42.3% (22)	81.1% (60)
Someone in the house owns a car	29.4% (47)	30.4% (17)	6.9% (3)	37.7% (72)	25.0% (13)	14.9% (11)
Number of people in house last night	6 [4, 8]	4 [3, 6]	3 [2, 4]	7 [5, 10]	4 [3, 6]	3 [2, 4]
Exercise						
Does not exercise	1.9% (3)	17.0% (9)	4.7% (2)	7.0% (13)	19.2% (10)	4.1% (3)
<5 hours a week	23.6% (37)	30.2% (16)	25.6% (11)	56.2% (105)	63.5% (33)	46.0% (34)
5–10 hours a week	61.8% (97)	45.3% (24)	48.8% (21)	30.5% (57)	13.5% (7)	44.6% (33)
>10 hours a week	12.7% (20)	7.6% (4)	20.9% (9)	6.4% (12)	3.9% (2)	5.4% (4)
Uses alcohol more than once a week	46.0% (64)	61.5% (32)	64.3% (27)	13.4% (22)	32.0% (16)	45.6% (31)
Currently smokes	77.9% (109)	66.7% (34)	24.4% (10)	73.5% (119)	51.0% (25)	5.9% (4)
BMI						
Underweight	21.4% (34)	5.5% (3)	0% (0)	28.0% (53)	7.7% (4)	8.1% (6)
Normal	51.6% (82)	38.2% (21)	58.1% (25)	34.9% (66)	36.5% (19)	58.1% (43)
Overweight	18.2% (29)	34.6% (19)	27.9% (12)	22.8% (43)	32.7% (17)	25.7% (19)
Obese	8.8% (14)	21.8% (12)	14.0% (6)	14.3% (27)	23.1% (12)	8.1% (6)

Notes: Presented as Median [Interquartile range] for continuous variables and % (n) for categorical variables.

Significant differences were observed in the lifestyle factors by Indigeneity, residence and gender. For example, over two thirds of Indigenous males were current smokers compared to a quarter of non-Indigenous males. The proportion of Indigenous females who smoked was slightly less than their male counterparts, but markedly higher than in non-Indigenous females among whom 6% smoked. In contrast, reported alcohol use was the highest among non-Indigenous males and females.

Overall, almost a quarter of urban Indigenous participants, both males and females, were affected with obesity, with this proportion considerably higher than for remote Indigenous and urban non-Indigenous counterparts. A third of urban Indigenous participants were in the overweight category, again higher than for remote Indigenous and urban non-Indigenous counterparts (see Table 1 for details). A quarter of remote Indigenous participants were in the underweight category, with a higher prevalence observed among females than males (Table 1).

For both males and females, apart from reduced HDL-c, all of the selected abnormal cardio-metabolic markers were significantly associated with BMI (*p* < 0.05) (see Table 2). A progressive increase in the prevalence of each abnormal marker was observed with increasing BMI category, except for hs-CRP among males. Elevated waist circumference was notable among females, present in half of those of normal weight and almost all (98.6–100%) of those in the overweight or obese categories. Among the Indigenous participants, elevated waist circumference was present in all females in the overweight and obese categories, as well as 59% and 75% of those with normal BMI in remote and urban areas, respectively (data not shown). Reduced HDL-c level was present in 56–79% of males and as many as 80 to 90% of females, irrespective of BMI category.

**Table 2:** Prevalence of abnormal cardio-metabolic markers by gender and BMI categories in Life Course Program wave 4 study participants.

Abnormal markers	Males (n = 259)					Females (n = 317)				
	Underweight	Normal	Overweight	Obese	P (Chi-sq)	Underweight	Normal	Overweight	Obese	P (Chi-sq)
Elevated waist cir^a^	0% (0)	4.0% (5)	48.3% (28)	100.0% (30)	<0.001	3.3% (2)	49.6% (59)	98.6% (70)	100.0% (41)	<0.001
Elevated triglycerides^b^	5.9% (2)	29.9% (35)	49.1% (26)	65.5% (19)	<0.001	1.8% (1)	13.0% (15)	30.0% (21)	35.0% (14)	<0.001
Reduced HDL-c^c^	55.9% (19)	58.8% (67)	66.0% (35)	79.3% (23)	0.17	81.8% (45)	78.1% (89)	91.4% (64)	92.3% (36)	<0.05
Elevated SBP or DBP^d^	11.1% (4)	15.5% (19)	21.4% (12)	51.6% (16)	<0.001	0.0% (0)	0.8% (1)	10.1% (8)	14.0% (6)	<0.001
Elevated HbA1c^e^	12.1% (4)	12.0% (14)	7.6% (4)	37.9% (11)	<0.001	12.7% (7)	6.1% (7)	23.2% (16)	30.0% (12)	<0.001
Elevated hs-CRP^f^	60.6% (20)	42.3% (47)	61.5% (32)	82.8% (24)	<0.001	30.9% (17)	44.7% (51)	84.3% (59)	92.5% (37)	<0.001
Number of abnormal markers										
0	21.9% (7)	17.0% (18)	8.2% (4)	0.0% (0)	<0.001^1^	13.5% (7)	12.4% (13)	0.0% (0)	0.0% (0)	<0.001^1^
1	25.0% (8)	23.6% (25)	16.3% (8)	7.4% (2)		51.9% (27)	25.7% (27)	4.8% (3)	0.0% (0)	
2	37.5% (12)	41.5% (44)	24.5% (12)	0.0% (0)		26.9% (14)	29.5% (31)	12.7% (8)	11.4% (4)	
3	15.6% (5)	12.3% (13)	26.5% (13)	14.8% (4)		7.7% (4)	24.8% (26)	39.7% (25)	25.7% (9)	
4	0.0% (0)	5.7% (6)	12.2% (6)	37.0% (10)		0.0% (0)	6.7% (7)	28.6% (18)	45.7% (16)	
5	0.0% (0)	0.0% (0)	10.2% (5)	29.6% (8)		0.0% (0)	1.0% (1)	7.9% (5)	17.1% (6)	
6	0.0% (0)	0.0% (0)	2.0% (1)	11.1% (3)		0.0% (0)	0.0% (0)	6.4% (4)	0.0% (0)	

Notes: Number of missing values for each item varies.

^1^Shows Probability for Fisher’s exact test using Monte Carlo estimation due to >20% of cells having an expected count of less than 5.

^a^Elevated waist circumference ≥94 cm – males; ≥80 cm – females.

^b^Elevated triglycerides (or drug treatment for elevated triglycerides) ≥150 mg/dL (1.7 mmol/L).

^c^Reduced High Density Lipoprotein Cholesterol (drug treatment for reduced HDL-c) <40 mg/dL (1.0 mmol/L) – males; <50 mg/dL (1.3 mmol/L) – females.

^d^Elevated blood pressure (or antihypertensive drug treatment) Systolic ≥130 and/or Diastolic ≥85 mm Hg.

^e^Elevated Glycated Haemoglobin (or drug treatment for diabetes) >=5.7% (39 mmol/mol).

^f^Elevated high sensitivity C-Reactive Protein >=3.0 μg/mL.

As observed for most individual markers, the cumulative number of abnormal cardio-metabolic markers was significantly positively associated with BMI category (*p* < 0.001). All of the males in the obese category and all of the females in the overweight and obese categories had at least one abnormal cardio-metabolic marker, with over 92% of males and over 88% of females affected with obesity having three or more. Of particular note was the prevalence of adverse cardio-metabolic profiles in the normal and underweight BMI categories. Among those of normal weight, almost one in five males and one in three females, and among the underweight, one in ten males and females had adverse cardio-metabolic profiles. Indigenous participants were more likely to have a higher number of abnormal cardio-metabolic markers than their non-Indigenous counterparts across all BMI categories (data not shown).

The findings from the logistic regression analysis, presented separately for males and females (see Table 3), showed that for each unit increase in BMI the odds of having adverse cardio-metabolic profile increased by 34% (AOR = 1.34, 95% CI [1.22–1.47]) among males and by 55% (AOR = 1.55, 95% CI [1.39–1.73]) among females, after taking into account Indigenous status/residency and other socio-economic factors. The associations between Indigenous status and residence and adverse cardio-metabolic profiles were different for males and females. Among males, the odds of having adverse cardio-metabolic profile were not significantly different for urban and remote Indigenous participants, but were significantly lower for urban non-Indigenous compared to urban Indigenous (AOR = 0.83, 95% CI [0.33–2.11] for remote Indigenous; and AOR = 0.08, 95% CI [0.02–0.35] for urban non-Indigenous). Among females, the odds of having adverse cardio-metabolic profile were significantly higher among remote Indigenous participants compared to urban Indigenous (AOR = 10.1, 95% CI [2.76–37.0]), but statistically not different for urban non-Indigenous (AOR = 0.65, 95% CI [0.14–3.04]). When exercise, alcohol intake and smoking were included in the model, none of these lifestyle factors were significantly associated with the outcome or affected the association between BMI and the outcome (data not shown). Thus, the models presented include adjustment for sociodemographic factors only.

**Table 3:** Logistic regression models predicting the probability of having adverse cardio-metabolic profile (3 or more cardio-metabolic risk indicators) for males and females (estimates and odds ratios).

	Males			Females		
Independent Variables	Odds Ratio	Pr > ChiSq	95% CI	Odds Ratio	Pr > ChiSq	95% CI
Body Mass Index	1.34	<0.001	1.22–1.47	1.55	<0.001	1.38–1.72
Indigeneity/residency						
Urban Indigenous [Ref]	1.00			1.00		
Remote Indigenous	0.83	0.699	0.32–2.10	10.10	0.001	2.75–37.0
Urban Non-Indigenous	0.08	0.001	0.01–0.35	0.65	0.582	0.13–3.04
Years of schooling						
<12 years [Ref]	1.00			1.00		
12 years	1.33	0.528	0.55–3.20	2.37	0.087	0.87–6.41
Main source of household income						
Social Security/other [Ref]	1.00			1.00		
Employment	0.80	0.595	0.34–1.83	1.09	0.885	0.33–3.53
Car ownership						
No car in the household [Ref]	1.00			1.00		
Owns a car	1.62	0.329	0.61–4.26	0.28	0.026	0.09–0.85
Someone in the household owns a car	2.36	0.064	0.95–5.82	0.88	0.789	0.34–2.23
Number of cases	213			251		
-2 Log Likelihood	186.074			171.945		

Notes: [Ref]- the reference category.

**Figure 1:**
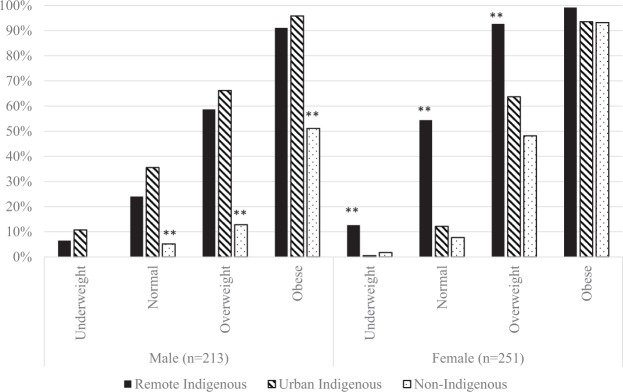
Average predicted probabilities of having 3 or more abnormal cardio-metabolic markers by BMI categories, Indigeneity, residence and gender. Notes: Based on Logistic Regression analysis, controlling for: education, source of income, and car ownership. Level of statistical significance: **p<0.001 (Reference category – Urban Indigenous).

For better understanding of the magnitude of the effect of BMI on adverse cardio-metabolic profile by Indigeneity and residence, Fig. 1 illustrates the average predicted probabilities of meeting adverse cardio-metabolic profile by BMI categories, based on the logistic regression analysis. Overall, the predicted probabilities of having an adverse cardio-metabolic profile were lower among males than females for all Indigeneity/residency groups and across all BMI categories. The only exception was among urban Indigenous participants in the overweight or obese categories, where the predicted probabilities of having an adverse cardio-metabolic profile were similar between males and females. Urban non-Indigenous males had the lowest predicted probabilities of all, with only 13% of those in the overweight category and 51% of those in the obese category having adverse cardio-metabolic profiles. On the other hand, remote Indigenous females had the highest predicted probability of multiple abnormal cardio-metabolic markers. Of note is that over half of those with normal BMI met adverse cardio-metabolic profiles, and almost all of those who were in the overweight or obese categories.

## Discussion

In this cohort of young adults, about 1 in 8 males and 1 in 7 females were affected with obesity, with the highest rates of obesity occurring among Indigenous males and females living in urban areas. Consistent with international literature our findings show that increasing BMI is positively associated with multiple abnormal cardio-metabolic markers^31,32^. The vast majority of both males and females affected with obesity had adverse cardio-metabolic profiles, with the exception of non-Indigenous males. However, a substantial proportion of Indigenous participants in the overweight and normal BMI categories also had adverse cardio-metabolic profiles, which may put Indigenous people at elevated risk for CVD. This may be linked to central adiposity, reflected in the high prevalence of elevated waist circumference particularly among Indigenous females.

Indigenous females living in remote areas had particularly higher prevalence of adverse cardio-metabolic profiles, with 3 or more abnormal cardio-metabolic markers present in almost all of those in the obese category, over ninety percent in the overweight category, over half in the normal BMI category and even 13 percent in the underweight category. Among males, there were few substantive differences between remote and urban Indigenous participants, and both were more likely to have multiple abnormal cardio-metabolic markers than urban non-Indigenous males across all BMI categories. The high prevalence of multiple abnormal cardio-metabolic markers overall is consistent with the findings of research by O’Dea and colleagues with young urban Indigenous people^12^. In their study, 45 percent of participants aged less than 35 and without diabetes had at least two cardiovascular risk factors.

The analysis of individual abnormal cardio-metabolic markers revealed that reduced HDL-c and elevated hs-CRP were the most prevalent markers in males and females, affecting substantial proportions of even those who were underweight or of normal weight. Previous research suggests reduced HDL-c is widespread among Indigenous people across the lifespan^13,33–36^. The reasons for this are not well understood. A high prevalence of infectious disease leading to chronic inflammation, dietary patterns that feature a high carbohydrate intake, and genetic factors are likely to contribute^34,35,37–39^. The most recent evidence suggests that HDL-c may be correlated with CVD risk only in healthy individuals^40^. Lifestyle changes including smoking recession, increased physical activity and weight loss have been suggested to increase HDL-c for improvement in cardiovascular health and reducing risk of disease^40,41^.

High levels of hs-CRP have been previously reported among Australian Indigenous populations^42^, especially among Indigenous females^43,44^. Possible explanations include psychosocial stress, a high prevalence of infectious disease due to poor quality housing and overcrowding^43^, as well as poor nutrition and inadequate physical activity, all linked to broader issues such as food insecurity, reduced access to education and economic opportunities and social inequality^42^. Some authors have suggested that central adiposity largely explains the elevated hs-CRP noted in many Indigenous people, but not the gender differences^43,44^. In other young adults, the implications of elevated hs-CRP for cardiovascular health remain unclear^45^. Further research is needed to understand the relationships between hs-CRP, BMI and CVD risk particularly in Indigenous populations, as this may shed light on new targets for therapy in the prevention of CVD.

Nevertheless, as weight, HDL-c and hs-CRP are all amenable to lifestyle modification, our findings are consistent with existing policy recommendations^46^ which promote lifestyle risk reduction as the primary focus of strategies to reduce cardiovascular risk. Prevention in childhood and adolescence should be the priority, with interventions tailored to cultural and social needs. It must be accompanied with broader strategies that address structural determinants of health inequalities, including lack of access to adequate housing, healthy food and opportunities for physical activities. Particular consideration must be given to Indigenous females in remote areas, given the markedly high prevalence of CVD markers observed in this group, even among those who were underweight or had a normal BMI. In other populations, there is sound evidence that metabolically-unhealthy normal-weight individuals are at substantially increased risk of CVD than metabolically healthy normal-weight individuals^47–50^. Further research is needed to understand the pathways underlying the early onset of risk in this group.

The main strength of the present study is that data were obtained by direct measurement of participants and using rigorous, standardized procedures, despite challenges posed by vast distances. However, there are some limitations. The study population is relatively small and while representative of the Indigenous people of the Top End of the Northern Territory, is not representative of the entire Indigenous population in Australia. The difficulty in obtaining reliable fasting blood samples limited our ability to use fasting plasma glucose and insulin levels in our analysis. Conversely, the rich data obtained allowed us to use alternative measures. The prevalence of obesity in this study was much lower than reported in the national statistics for this age group; however, this might be explained by the under-coverage of remote areas in the 2012–13 Australian Aboriginal and Torres Strait Islander Health Survey^9^.

## Conclusion

Young adulthood is a critical time when behaviours are formed that shape lifelong health. The alarmingly high prevalence of adverse cardio-metabolic profiles found among young Indigenous males and females in this study indicates an urgent need for programs aimed at risk mitigation, targeting the priority areas of unhealthy weight and resultant inflammation. Success will be dependent upon broader policies that address structural constraints affecting health behaviours and engage Indigenous young people, to ensure strategies are culturally relevant.

## Data Availability

All data is stored confidentially and although not freely available in the public domain due to conditions imposed by the Human Research Ethics Committee. However, data is available on request and specific proposals for collaboration are welcomed. Collaborations are established through formal agreement with the steering committee.
